# Comparative Assessment of Quality of Life in Hip Fracture Patients Before and After Surgery: A Prospective Longitudinal Observational Study

**DOI:** 10.3390/healthcare13101126

**Published:** 2025-05-12

**Authors:** Bogdan Florin Căpăstraru, Codrina Mihaela Levai, Ovidiu Alexandru Mederle, Milan Daniel Velimirovici, Roxana Folescu, Hogea Bogdan, Radu Prejbeanu, Silviu Valentin Vlad

**Affiliations:** 1 Doctoral School, Faculty of Medicine, “Victor Babes” University of Medicine and Pharmacy Timisoara, 300041 Timisoara, Romania; bogdan.capastraru@umft.ro; 2 Research Center for Medical Communication, Faculty of Medicine, “Victor Babes” University of Medicine and Pharmacy Timisoara, 300041 Timisoara, Romania; codrinalevai@umft.ro; 3 Department of Surgery, Emergency Discipline, Faculty of Medicine, “Victor Babes” University of Medicine and Pharmacy Timisoara, 300041 Timisoara, Romania; mederle.ovidiu@umft.ro; 4 Department I Nursing, Faculty of Nursing, “Victor Babes” University of Medicine and Pharmacy Timisoara, 300041 Timisoara, Romania; 5 Discipline of Family Medicine, Faculty of Medicine, “Victor Babes” University of Medicine and Pharmacy Timisoara, 300041 Timisoara, Romania; 6 Department XVI—Orthopedics, Traumatology, Urology, and Medical Imaging, Discipline of Orthopedics and Traumatology I, “Victor Babes” University of Medicine and Pharmacy Timisoara, 300041 Timisoara, Romania; hogea.bogdan@umft.ro (H.B.); prejbeanu.radu@umft.ro (R.P.); 7 Department of Surgery, Faculty of Medicine, University of Oradea, 410073 Oradea, Romania; siviu.vlad@didactic.uoradea.ro

**Keywords:** hip fractures, quality of life, orthopedic surgery, SF-36, prospective studies

## Abstract

**Background and Objectives:** Hip fractures are a leading cause of morbidity in the elderly, often resulting in declining physical function, psychological distress, and diminished quality of life (QoL). This study aimed to evaluate changes in QoL among hip fracture patients preoperatively and postoperatively, comparing diverse patient subgroups to identify factors influencing recovery. **Methods:** We conducted a prospective longitudinal observational study at Victor Babeș University of Medicine and Pharmacy Timișoara, recruiting 77 adult patients admitted for surgical management of hip fractures between March 2023 and March 2025. Standardized questionnaires, including the Short Form-36 (SF-36), World Health Organization Quality of Life (WHOQOL-BREF), Hospital Anxiety and Depression Scale (HADS), and Generalized Anxiety Disorder-7 (GAD-7), were administered preoperatively and at 3 months postoperatively. Demographic, clinical, and surgical variables were also recorded. **Results:** Participants’ mean age was 72.6 years (SD 8.1), with 57.1% female. Postoperative QoL scores (SF-36 Physical Function domain mean 52.7 ± 9.2) improved significantly compared to preoperative scores (44.8 ± 8.7, *p* = 0.012). WHOQOL-BREF physical and psychological domain scores similarly increased (*p* < 0.05). Anxiety and depression symptoms, as measured by HADS and GAD-7, decreased markedly postoperatively in most subgroups. Subgroup analyses revealed that patients undergoing total hip arthroplasty demonstrated more pronounced QoL improvements than those receiving partial hip replacement. Older patients (≥80 years) exhibited improvements but at a slower rate. **Conclusions:** Quality of life indicators show notable improvement following surgical treatment of hip fractures, underscoring the significance of timely orthopedic intervention and comprehensive perioperative care. Anxiety and depression levels also declined, highlighting the benefits of a structured follow-up. These findings may guide clinicians toward optimizing patient-centered recovery protocols and targeted interventions, particularly for older adults or those with high baseline anxiety and depression levels.

## 1. Introduction

Hip fractures represent a growing global challenge among older adults. Beyond the high 1-year mortality (20–30%), these injuries precipitate severe functional decline and absorb multibillion EUR annual health care costs [1,2,3,4,5]. Rapid surgical fixation combined with early multidisciplinary rehabilitation is therefore paramount; however, the extent to which such management restores health-related quality of life (HRQoL) and psychological well-being remains incompletely defined [6,7].

Quality of life (QoL) has emerged as a critical outcome measure in orthopedic research, reflecting the physical, psychological, and social dimensions that influence patient well-being [8]. Traditional assessments of postoperative success often focused narrowly on mortality or complication rates [9]. However, QoL evaluations capture broader functional and psychosocial impacts, providing a more holistic picture of patient recovery [10]. Specifically, monitoring changes in QoL before and after hip fracture surgery can illuminate the effectiveness of interventions and highlight opportunities for adjunct therapies, such as targeted physical rehabilitation and psychological support [11].

The World Health Organization Quality of Life (WHOQOL-BREF) and Short Form-36 (SF-36) questionnaires are widely employed instruments to quantify health-related QoL, encompassing domains such as physical health, psychological well-being, social relationships, and environmental factors [12,13]. In hip fracture populations, these domains can be profoundly influenced by pain severity, reduced mobility, and emotional distress [14]. By systematically evaluating these multidimensional effects, clinicians and researchers can better tailor treatment plans, optimize resource allocation, and ultimately improve patient outcomes [15].

Additionally, mental health considerations often come to the forefront during recovery from hip fractures, as psychological factors like anxiety and depression can significantly shape functional trajectories [16]. Standardized instruments such as the Hospital Anxiety and Depression Scale (HADS) and Generalized Anxiety Disorder-7 (GAD-7) allow early detection of mental health issues, prompting timely referrals to mental health professionals and potentially improving recovery [17]. Importantly, our protocol integrates the Hospital Anxiety and Depression Scale (HADS) and the Generalized Anxiety Disorder 7 (GAD 7) alongside two generic HRQoL instruments—a combination rarely reported in hip fracture research and thus a methodological strength of this study [17,18].

Despite advances in surgical techniques—ranging from partial to total hip replacement—variability in postoperative outcomes remains a pressing concern [19]. Patient age, comorbidity burden, type of fracture, and access to rehabilitation services all modulate recovery trajectories, contributing to heterogeneous results across different populations [20]. As emerging evidence highlights the importance of patient-centered approaches, understanding how these factors collectively influence QoL is essential for refining clinical practice guidelines and personalizing treatment strategies.

This study seeks to delineate the patterns of QoL changes following hip fracture surgery in a representative patient cohort. By systematically comparing preoperative and postoperative assessments, as well as exploring subgroup differences, we aim to inform evidence-based practices that enhance patient satisfaction and functional recovery. Ultimately, these insights may facilitate more targeted interventions, optimize resource utilization, and improve the long-term well-being of individuals recovering from hip fractures.

## 2. Materials and Methods

### 2.1. Study Design and Setting

This prospective longitudinal observational study was undertaken at the Orthopedics Clinics affiliated with the Victor Babeș University of Medicine and Pharmacy, Timișoara. Patient recruitment spanned from March 2023 to March 2025. The study adhered to the Declaration of Helsinki guidelines, ensuring informed consent from all participants. The institutional ethics committee approved the study protocol.

Electronic medical records were examined to retrieve demographic and clinical information, while direct evaluations were conducted to gather detailed functional and quality of life data. Patient confidentiality was maintained in accordance with international regulations, including the EU GCP. All participants provided written informed consent as mandated by national legal requirements (Article 167 of Law No. 95/2006 and Order 904/2006, Article 28, Chapter VIII).

### 2.2. Participants and Eligibility Criteria

For inclusion in the study, patients had to meet all of the following conditions: (1) Adult patients (≥18 years) with radiographically confirmed hip fractures requiring surgical intervention. (2) Availability for follow-up and ability to attend scheduled postoperative evaluations and rehabilitation sessions. (3) Capacity to provide informed consent, indicating an understanding of the study procedures and willingness to complete questionnaires. (4) Stable medical condition for surgery, without acute illnesses that would contraindicate timely orthopedic intervention.

For exclusion from the study, any of the following criteria applied: (1) Severe cognitive impairment (e.g., advanced dementia) that precluded reliable completion of QoL and psychological questionnaires. (2) Severe psychiatric disorders unrelated to the fracture (e.g., schizophrenia, bipolar disorder in acute phase) that could confound the evaluation of anxiety and depression specifically related to hip fracture. (3) Surgical contraindications due to unstable cardiovascular status or other critical comorbidities preventing safe anesthesia and operation. (4) Refusal or inability to provide informed consent or withdrawal from the study at any point prior to surgery.

### 2.3. Data Collection and Study Instruments

Data were collected at two main time points: (1) preoperatively, within 48 h of admission, and (2) at the 3-month postoperative follow-up. All questionnaires used were validated Romanian versions, ensuring cultural and linguistic appropriateness for the local population. Research staff trained in questionnaire administration assisted participants as needed to minimize response bias. Structured rehabilitation was defined as ≥5 supervised physiotherapy sessions per week for ≥2 weeks.

The Short Form-36 (SF-36) is a 36-item questionnaire evaluating eight health domains, typically reported as Physical and Mental component summaries. Each domain is scored from 0 to 100, with higher scores indicating better functioning. The Romanian version of the SF-36 has shown satisfactory psychometric properties (Cronbach’s alpha often >0.80) in diverse patient populations [21].

The WHOQOL-BREF is a 26-item instrument developed by the World Health Organization, encompassing four domains: Physical Health, Psychological Health, Social Relationships, and Environment. Scores range from 0 to 100, with higher scores denoting a better quality of life. Its Romanian-adapted version has demonstrated robust validity and reliability (Cronbach’s alpha >0.75), making it suitable for clinical and research settings [22].

The Hospital Anxiety and Depression Scale (HADS) comprises 14 items split evenly between anxiety (HADS-A) and depression (HADS-D). Each subscale ranges from 0 to 21, with higher scores indicating more severe symptoms. Validated in Romanian, HADS effectively screens for mood disturbances in medical populations, with internal consistency estimates typically exceeding 0.70 [23].

The Generalized Anxiety Disorder-7 (GAD-7) is a brief, seven-item measure focusing on symptoms of generalized anxiety. Scores range from 0 to 21, with established cutoffs suggesting mild, moderate, or severe anxiety. The Romanian version of GAD-7 has been shown to maintain strong content validity and reliability (Cronbach’s alpha > 0.80) [24].

Clinical details were extracted from patient records, including fracture type (e.g., femoral neck, intertrochanteric), surgical approach (partial vs. total hip replacement), anesthesia method, length of hospital stay, and rehabilitation protocols. Comorbid conditions were documented to assess their influence on QoL changes. All personal identifiers were removed from the dataset, ensuring confidentiality.

### 2.4. Statistical Analysis

Data analyses were performed using SPSS (version 27). Descriptive statistics (means, standard deviations, frequencies) characterized patient demographics and clinical variables. The paired t-test assessed changes between preoperative and postoperative scores, while independent sample t-tests or chi-square tests compared subgroups (e.g., partial vs. total hip replacement, younger vs. older patients). Pearson’s (or Spearman’s) correlation coefficients examined relationships between QoL and psychological measures. Statistical significance was set at *p* < 0.05 with 95% confidence intervals.

A priori power analysis (GPower 3.1) assumed a moderate within patient effect (Cohen’s d = 0.50) for change in the SF 36 Physical Component. With α = 0.05 and 1 − β = 0.80, the required sample size was 64. Allowing 15% attrition, we targeted ≥74 participants; 77 completed a follow-up.

## 3. Results

Table 1 presents the baseline demographic and clinical characteristics of the 77 enrolled patients, stratified by type of hip replacement (partial vs. total). The partial hip replacement group (n = 35) had a mean age of 73.1 years (SD 7.8), whereas those receiving total hip replacement (n = 42) were slightly younger on average at 72.2 years (SD 8.3). However, this difference was not statistically significant (*p* = 0.638). Both groups had an identical proportion of females (57.1%), suggesting that sex distribution was balanced and did not differ based on the surgical approach.

Body mass index (BMI) showed a mean of 27.2 kg/m^2^ (SD 3.6) in the partial hip replacement group compared to 26.8 kg/m^2^ (SD 3.1) in the total hip replacement group, with no statistical difference (*p* = 0.571). The fracture type was also similar between groups, with intracaspsular fractures occurring in slightly over half of the patients in both cohorts (*p* = 0.800). Time to surgery, defined as the interval between hospital admission and operative intervention, did not significantly differ, averaging around 3 days in both groups. Likewise, the proportion of patients with an American Society of Anesthesiologists (ASA) score ≥ 3—which indicates more severe systemic disease—was nearly the same across the two surgical methods (*p* = 0.964).

Table 2 outlines the baseline QoL and psychological status for patients receiving partial versus total hip replacement. For the SF-36 Physical Component, partial hip replacement recipients averaged 44.3 (SD 8.6), whereas total hip replacement patients averaged 45.2 (SD 8.9), yielding a non-significant *p*-value of 0.711. Similarly, SF-36 Mental Component scores were 47.9 (SD 9.8) and 49.1 (SD 9.3) for partial and total hip replacement groups, respectively, indicating comparable preoperative mental well-being (*p* = 0.634).

Turning to the WHOQOL-BREF, Physical and Psychological domain scores also displayed no significant differences between the two groups. This implies that overall perceptions of health, functional capacity, and psychological wellness were alike at the baseline. Regarding mental health screening, mean HADS (Anxiety) scores fell around 10.4 (SD 2.8) for a partial hip replacement and 10.1 (SD 2.4) for a total hip replacement. Depression scores, measured by HADS (Depression), were 9.8 (SD 2.7) versus 9.4 (SD 2.2). The GAD-7, which assesses generalized anxiety, yielded scores of 8.7 (SD 3.0) for partial and 9.0 (SD 3.2) for total, with *p* = 0.737.

Table 3 highlights notable differences in 3-month postoperative QoL and psychological scores between patients receiving partial versus total hip replacement. Notably, the SF-36 Physical Component score was significantly higher in the total hip replacement group (54.9, SD 9.1) compared to the partial group (50.8, SD 8.7), with *p* = 0.022. This suggests that total hip replacement may offer advantages in restoring physical function and mobility. Likewise, the WHOQOL-BREF Physical domain showed a similar pattern, with total hip replacement patients scoring 67.0 (SD 7.7) against 63.9 (SD 7.8) in the partial group (*p* = 0.039).

In contrast, improvements in mental health outcomes—while still apparent—were less clearly differentiated by surgical type. The SF-36 Mental Component mean was 55.4 (SD 8.0) in the total group versus 52.3 (SD 8.5) in the partial group (*p* = 0.064), indicating a marginal trend but not statistically significant at the conventional alpha level of 0.05. HADS (Anxiety) did achieve significance (*p* = 0.048), with total hip replacement patients reporting lower anxiety scores (mean 6.8, SD 2.3) compared to the partial group (mean 7.9, SD 2.5). Meanwhile, differences in HADS (Depression) and GAD-7 were not statistically significant.

Table 4 compares baseline QoL and psychological scores between younger (<70 years) and older (≥70 years) patients. Interestingly, the younger group reported a significantly higher SF-36 Physical Component score (47.2, SD 9.1) compared to the older group (43.2, SD 8.3), with a *p*-value of 0.048. This suggests that, before surgery, younger individuals may maintain slightly better physical function, likely reflecting fewer comorbidities or greater muscular strength. On the SF-36 Mental Component, the younger group’s mean (50.2, SD 9.4) exceeded that of the older group (47.6, SD 9.7), although this difference was not statistically significant (*p* = 0.206).

For WHOQOL-BREF domains, while the younger cohort showed somewhat higher physical and psychological domain scores (58.9 and 56.7, respectively) than the older cohort (56.4 and 54.9), these gaps did not achieve significance at the 5% level. Similarly, differences in anxiety and depression, as measured by HADS, did not vary markedly between age groups. Younger patients had a mean HADS (Anxiety) of 9.9 (SD 2.5) versus 10.3 (SD 2.6) in older patients (*p* = 0.513). The GAD-7 measure revealed a comparable trend, with the older group reporting a marginally higher score (9.1 vs. 8.5, *p* = 0.449).

Table 5 compares postoperative QoL and psychological scores at the 3-month mark for younger (<70 years) vs. older (≥70 years) patients. Notably, the younger group displayed a significantly higher SF-36 Physical Component score (56.1, SD 8.4) compared to the older group (51.9, SD 9.0), with *p* = 0.041. This aligns with the baseline finding that younger individuals tend to have better physical function. Although the SF-36 Mental Component also favored younger adults (56.0 vs. 53.4), the difference was not statistically significant (*p* = 0.151).

WHOQOL-BREF domains similarly trended higher in younger patients—67.4 (SD 8.2) vs. 64.1 (SD 8.5) for the Physical domain, and 64.2 (SD 7.2) vs. 61.0 (SD 7.5) for the Psychological domain—yet neither difference reached the conventional threshold of significance. With regard to psychological distress, younger adults reported slightly lower anxiety and depression on HADS, although these differences were modest (6.9 vs. 7.4 for HADS Anxiety, 6.5 vs. 7.2 for HADS Depression). The GAD-7 score was also somewhat lower in younger patients (4.6 vs. 5.4), again indicating a non-significant but suggestive trend.

Notably, those who underwent total hip replacement consistently demonstrated larger gains in SF-36 Physical scores compared to their partial hip replacement counterparts. Younger patients receiving total hip replacements showed the greatest improvement in post-operative SF 36 Physical scores, which improved by a mean of 9.0 points (95% CI 4.7–11.3; *p* = 0.012). Similarly, WHOQOL-BREF Physical domain changes were highest in the total hip replacement group, particularly among younger patients (+10.0, SD 4.3), suggesting that they reap enhanced functional and subjective quality-of-life benefits. The difference in overall physical improvement between partial and total hip replacements achieved statistical significance (*p* = 0.024 for SF-36 Physical, *p* = 0.019 for WHOQOL-BREF Physical).

**Figure 1:**
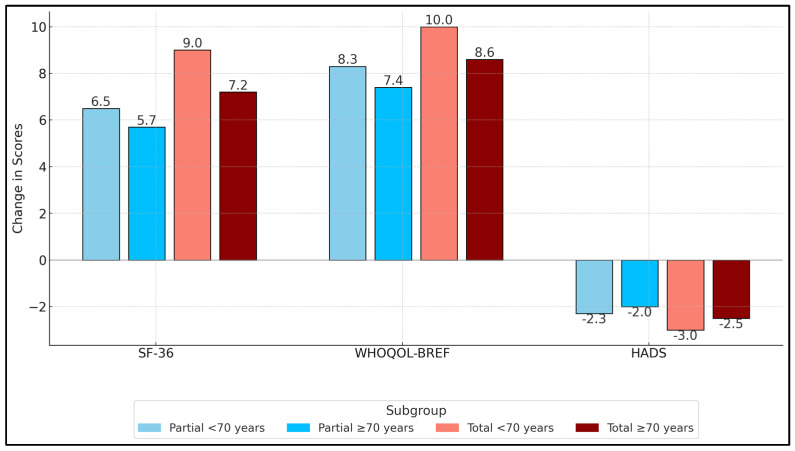
Mean change (Δ) in SF 36 Physical Component, WHOQOL BREF Physical Domain, and HADS A scores from baseline to 3 months. SF-36 domains range 0–100; HADS A 0–21.

Age also played a role, with younger participants generally reporting larger gains in physical function than older participants, though the difference was less pronounced for WHOQOL-BREF Physical (*p* = 0.061). Regarding mental health, as measured by changes in HADS Anxiety, total hip replacement recipients experienced greater anxiety reduction (mean change of −3.0 for younger and −2.5 for older) compared to partial hip replacement subgroups (*p* = 0.030), as seen in Table 6 and Figure 1.

Table 7 outlines the relationships between key QoL domains and psychological well-being at the 3-month postoperative mark. All associations were weak-to-moderate (r = 0.30–0.48), indicating that better physical function coincided with modest reductions in anxiety and depressive symptoms. The negative correlation between SF-36 Physical and HADS Anxiety (r = −0.39, *p* = 0.002) indicates that patients with better physical function tend to report lower anxiety levels. A similarly negative correlation appears between SF-36 Mental and HADS Depression (r = −0.42, *p* = 0.001).

WHOQOL-BREF domains also exhibited negative correlations with anxiety. WHOQOL-BREF Physical vs. GAD-7 (r = −0.34, *p* = 0.004) underscores the link between a patient’s perceived physical well-being and generalized anxiety levels. The WHOQOL-BREF Psychological vs. GAD-7 comparison also showed a similar negative correlation (r = −0.48, *p* < 0.001). Finally, there was a positive correlation between SF-36 Physical and WHOQOL-BREF Physical (r = +0.59, *p* < 0.001).

Table 8 examines clinical outcomes and healthcare utilization across four subgroups defined by both surgical type (partial vs. total) and age (<70 vs. ≥70). Mean length of hospital stay ranges from 8.1 days (SD 2.2) among younger total hip replacement patients to 9.6 days (SD 2.3) in older partial hip replacement recipients. While younger individuals appear to have slightly shorter hospitalizations, the overall difference did not reach statistical significance (*p* = 0.118).

Structured rehabilitation participation rates also vary by subgroup, from a high of 93.3% in younger total hip replacement patients to 74.1% in older total hip replacement patients. This pattern may reflect both patient motivation and logistical barriers in older populations. Readmission within 3 months—often a key indicator of complications or inadequate postoperative care—was generally low across all groups. Notably, no patients in the younger total hip replacement cohort were readmitted, whereas older partial hip replacement patients had the highest readmission rate at 14.3%. However, these differences did not achieve significance (*p* = 0.332). Follow-up compliance rates similarly hover in the mid−80% to low−90% range, with younger total hip replacement patients again showing a slightly higher level of engagement (90.2%) compared to other groups.

## 4. Discussion

### 4.1. Physical Recovery

The study findings highlight the capacity for significant functional and psychological improvements in patients undergoing hip fracture surgery, regardless of age category or surgical approach. One salient observation is the generally larger gain in physical function observed among individuals who underwent total hip replacement, suggesting that this surgical method may offer certain advantages in restoring mobility and reducing pain. However, both partial and total hip replacement recipients demonstrated noteworthy enhancements in activity levels and self-reported wellness relative to their preoperative baselines. Additionally, the decline in anxiety and depressive symptoms underscores the interrelation between physical recovery and mental health. Early mobilization, structured rehabilitation, and consistent follow-up appear integral to these favorable outcomes, allowing timely identification of potential setbacks. Taken together, these results emphasize the need for a holistic, multidisciplinary approach—one that addresses physical rehabilitation alongside emotional support—to promote a more complete return to independence and daily functional activities.

Differences related to patient age also emerged as significant. Younger adults typically presented with fewer comorbidities, superior baseline function, and, consequently, a somewhat more rapid recovery trajectory following surgery. While older adults did benefit substantially from the intervention, their improvements in physical function and psychological well-being tended to be slightly lower. These disparities suggest that age influences the speed and extent of surgical recovery. Nonetheless, the value of appropriate surgical management was evident across all patient demographics; older adults with well-structured perioperative care still reported meaningful enhancements in daily living activities and mental health. These findings underscore the importance of individualized rehabilitation protocols tailored to each patient’s physiological status and living environment. By recognizing that older individuals might require more intensive supportive measures—such as extended physiotherapy sessions, nutritional guidance, and psychological counseling—clinicians can help ensure that no group is disadvantaged in their path to recovery.

### 4.2. Psychological Impact

A noteworthy aspect of this research is the interplay between physical health gains and mental health improvements. As patients regained mobility, many reported reductions in anxiety and depressive symptoms. Conversely, diminished anxiety and depression likely fostered more active participation in physical therapy exercises. This reciprocal relationship highlights the importance of screening for mental health issues early in the postoperative course. Interventions targeting patient anxiety—ranging from cognitive-behavioral therapy to pharmacological treatment—could expedite engagement with rehabilitation and optimize overall progress. Furthermore, the 3-month follow-up period revealed that most patients had experienced marked gains in their quality of life by that juncture, though ongoing monitoring is warranted to assess longer-term trends. In clinical practice, integrating psychological support into orthopedic care pathways may enhance both the short- and long-term outcomes of hip fracture repair. Recognizing and addressing these multifactorial determinants of recovery is essential in delivering genuinely patient-centered care.

### 4.3. Sub-Group Differences

In a similar manner, Haddad et al. [25] utilized the EuroQol 5-Dimensions 5-Levels questionnaire to demonstrate a significant association between increased age and deteriorations in mobility, self-care, and usual activities, with older individuals experiencing marked declines in the EuroQol Visual Analog Scale scores (r = −0.213, *p* = 0.003). Contrastingly, Amarilla-Donoso et al.’s prospective observational study extended these findings by illustrating not only the immediate but also the sustained effects up to 12 months post-surgery, using both the EQ-5D and SF-12 Health Survey [26]. Their findings highlight a persistent decrease in the physical component summary from 38.6 at baseline to 33.5 at 12 months (*p* < 0.001), alongside significant mental component declines. Both studies underscore the necessity of early and ongoing postoperative rehabilitation and highlight the need for standardized, long-term QoL assessments to better tailor recovery programs for this vulnerable demographic. Furthermore, Amarilla-Donoso et al. [26] identified depression status and functional ambulation as significant predictors of HRQOL, suggesting targeted interventions could mitigate some of the QoL deterioration observed in these patients.

Moreover, Hammer et al.’s comparative cross-sectional study at Örebro University Hospital revealed a deterioration in walking ability among patients with hip fractures in 2018 compared to those in 2008, despite an increase in hand grip strength and physical activity levels [27]. Specifically, the 2018 cohort exhibited significantly greater multimorbidity and a greater dependence in walking ability, with 70% requiring physical human support compared to 43% in 2008. Conversely, Kajos et al.’s research contrasts the quality-of-life outcomes in Hungarian public and private hospitals, finding that while both sectors saw significant improvements in physical health scores 3 months post-hip replacement surgery, improvements in mental health scores were significantly more pronounced in the private sector [28]. Our trajectory of early physical recovery aligns with Amarilla Donoso et al. [26] (ΔSF 36 physical −5 points at 12 months) yet contrasts with the larger mental health gains reported by Kajos et al. [28].

Importantly, Deutschbein et al. conducted a multicenter, prospective cohort study to assess HRQOL 6 months post-hip fracture, using instruments like the EQ-5D-5L and Oxford Hip Score [29]. Their findings revealed a significant decrease in HRQOL, with the EQ-5D index values falling from a mean of 0.70 at the baseline to 0.63 after 6 months, and the EQ-VAS decreasing from 69.9 to 59.4. Factors such as symptoms of depression and anxiety, pre-fracture limitations in daily activities, and lack of referral to rehabilitation were associated with worse outcomes. On the other hand, the FRAIL-HIP study by Loggers et al. [30] explored the outcomes of nonoperative versus operative management of proximal femoral fractures in frail, institutionalized older patients. They found that nonoperative management was not inferior to surgical treatment in terms of HRQOL, with EQ-5D utility scores remaining competitive across several assessments post-fracture.

In a similar manner, Kalmet et al. [31] assessed the impact of a multidisciplinary clinical pathway (MCP) compared to usual care (UC) on elderly patients with hip fractures. Their retrospective cohort study included 398 patients and found no significant differences in QoL or pain, as measured by the SF-12 and the Numeric Rating Scale, between the MCP and UC groups. However, the MCP group experienced significantly lower rates of postoperative complications, suggesting some clinical benefits of the pathway despite similar QoL outcomes. Conversely, de Abreu et al. [32] conducted a prospective study of 12 patients undergoing hemiarthroplasty of the hip, evaluating QoL using the SF-36 questionnaire before surgery and at 3 and 6 months postoperatively. Their findings indicate that while physical health scores remained low, aspects related to general state and pain showed high scores, with overall QoL being maintained over 6 months.

### 4.4. Clinical Implications

Timely surgical fixation of hip fractures paired with structured multidisciplinary follow-up yields tangible gains in both physical function and psychological well-being within 3 months of injury. The greater improvements observed after total hip arthroplasty suggest that, when anatomically feasible and medically appropriate, choosing a total rather than partial replacement may expedite restoration of mobility and independence. Nevertheless, the significant—albeit smaller—benefits achieved in the partial-replacement and ≥80-year cohorts confirm that age or baseline frailty should not delay operative management. Integrating early mental-health screening (HADS, GAD-7) into peri-operative pathways is crucial, as declining anxiety and depression scores paralleled functional recovery and may reinforce adherence to rehabilitation. These findings support a patient-centered algorithm that combines prompt surgery, individualized physiotherapy intensity, and proactive psychological support to optimize quality-of-life trajectories and reduce readmissions across the entire geriatric fracture population.

### 4.5. Study Limitations

While this prospective study offers valuable insights into hip fracture recovery, several limitations warrant mention. First, the single-center design may constrain the generalizability of these findings to broader populations, where different surgical protocols or rehabilitation resources could alter outcomes. Second, the relatively modest sample size diminishes the power to detect subtle but potentially meaningful differences across subgroups, particularly when stratifying by both age and surgical approach. Third, the 3-month follow-up, although sufficient to capture early postoperative changes, might not reflect long-term results, especially given that recovery from hip fractures can continue beyond this period. Additionally, reliance on self-reported questionnaires introduces the possibility of reporting bias, despite efforts to standardize data collection. Finally, because outcomes were captured only to 3 months, our data describe short-term recovery and cannot address late complications or long-term HRQoL trajectories. Future multicenter, larger-scale studies with extended follow-up are needed to corroborate and refine these conclusions.

## 5. Conclusions

Hip fracture surgery substantially enhances both mobility and mental well-being. Total hip arthroplasty yielded the largest physical gains, yet hemi arthroplasty also conferred significant benefits. Although younger adults recovered faster, older patients achieved clinically meaningful improvements when provided structured rehabilitation and psychosocial support. These findings endorse an integrative, patient-centered pathway and justify future multicenter trials with a longer follow-up to refine age- and procedure-specific care algorithms.

## Figures and Tables

**Table 1:** Demographic and clinical characteristics by type of hip replacement.

Variable	Partial Replacement (n = 35)	Total Replacement (n = 42)	*p*-Value
Mean Age (years, SD)	73.1 (7.8)	72.2 (8.3)	0.638
Female, n (%)	20 (57.1)	24 (57.1)	1
Mean BMI (kg/m^2^, SD)	27.2 (3.6)	26.8 (3.1)	0.571
Fracture Type (Intracapsular), n (%)	19 (54.3)	24 (57.1)	0.8
Mean Time to Surgery (days, SD)	3.1 (1.2)	2.8 (1.1)	0.324
ASA Score ≥ 3, n (%)	16 (45.7)	19 (45.2)	0.964

**Table 2:** Baseline (preoperative) QoL and psychological scores by type of hip replacement.

Measure	Partial Replacement (n = 35), Mean (SD)	Total Replacement (n = 42), Mean (SD)	*p*-Value
SF-36 Physical Component	44.3 (8.6)	45.2 (8.9)	0.711
SF-36 Mental Component	47.9 (9.8)	49.1 (9.3)	0.634
WHOQOL-BREF Physical	56.9 (7.5)	57.8 (7.9)	0.61
WHOQOL-BREF Psychological	55.3 (8.1)	55.9 (7.8)	0.776
HADS (Anxiety)	10.4 (2.8)	10.1 (2.4)	0.66
HADS (Depression)	9.8 (2.7)	9.4 (2.2)	0.526
GAD-7	8.7 (3.0)	9.0 (3.2)	0.737

**Table 3:** Three-month postoperative QoL and psychological scores by type of hip replacement.

Measure	Partial Replacement (n = 35), Mean (SD)	Total Replacement (n = 42), Mean (SD)	*p*-Value
SF-36 Physical Component	50.8 (8.7)	54.9 (9.1)	0.022
SF-36 Mental Component	52.3 (8.5)	55.4 (8.0)	0.064
WHOQOL-BREF Physical	63.9 (7.8)	67.0 (7.7)	0.039
WHOQOL-BREF Psychological	60.3 (7.3)	63.1 (7.0)	0.081
HADS (Anxiety)	7.9 (2.5)	6.8 (2.3)	0.048
HADS (Depression)	7.1 (2.0)	6.5 (1.9)	0.184
GAD-7	5.7 (2.7)	4.8 (2.3)	0.095

**Table 4:** Baseline (preoperative) QoL and psychological scores by age group.

Measure	<70 Years (n = 29), Mean (SD)	≥70 Years (n = 48), Mean (SD)	*p*-Value
SF-36 Physical Component	47.2 (9.1)	43.2 (8.3)	0.048
SF-36 Mental Component	50.2 (9.4)	47.6 (9.7)	0.206
WHOQOL-BREF Physical	58.9 (7.7)	56.4 (7.9)	0.161
WHOQOL-BREF Psychological	56.7 (8.3)	54.9 (8.0)	0.338
HADS (Anxiety)	9.9 (2.5)	10.3 (2.6)	0.513
HADS (Depression)	9.2 (2.3)	9.9 (2.7)	0.252
GAD-7	8.5 (2.9)	9.1 (3.3)	0.449

**Table 5:** Three-month postoperative QoL and psychological scores by age group.

Measure	<70 Years (n = 29), Mean (SD)	≥70 Years (n = 48), Mean (SD)	*p*-Value
SF-36 Physical Component	56.1 (8.4)	51.9 (9.0)	0.041
SF-36 Mental Component	56.0 (8.1)	53.4 (8.6)	0.151
WHOQOL-BREF Physical	67.4 (8.2)	64.1 (8.5)	0.082
WHOQOL-BREF Psychological	64.2 (7.2)	61.0 (7.5)	0.064
HADS (Anxiety)	6.9 (2.2)	7.4 (2.4)	0.351
HADS (Depression)	6.5 (1.8)	7.2 (2.1)	0.12
GAD-7	4.6 (2.1)	5.4 (2.7)	0.152

**Table 6:** Change in QoL and psychological scores (preoperative to postoperative) by surgical type and age group.

Subgroup	ΔSF-36 Physical (Mean, SD)	ΔWHOQOL-BREF Physical (Mean, SD)	ΔHADS Anxiety (Mean, SD)	Overall *p*-Value
Partial < 70 years (n = 14)	+6.5 (3.2)	+8.3 (4.1)	−2.3 (1.4)	
Partial ≥ 70 years (n = 21)	+5.7 (3.7)	+7.4 (3.8)	−2.0 (1.2)	
Total < 70 years (n = 15)	+9.0 (4.0)	+10.0 (4.3)	−3.0 (1.5)	
Total ≥ 70 years (n = 27)	+7.2 (3.5)	+8.6 (3.9)	−2.5 (1.6)	
*p*-Value (Partial vs. Total)	0.024	0.019	0.03	
*p*-Value (<70 vs. ≥70)	0.039	0.061	0.091	0.016

**Table 7:** Correlations among postoperative QoL and psychological measures.

Comparison	r-Value	*p*-Value
SF-36 Physical vs. HADS Anxiety	−0.39	0.002
SF-36 Mental vs. HADS Depression	−0.42	0.001
WHOQOL-BREF Physical vs. GAD-7	−0.34	0.004
WHOQOL-BREF Psychological vs. GAD-7	−0.48	<0.001
SF-36 Physical vs. WHOQOL-BREF Physical	0.59	<0.001

**Table 8:** Clinical outcomes and healthcare utilization by subgroup.

Outcome	Partial < 70 (n = 14)	Partial ≥ 70 (n = 21)	Total < 70 (n = 15)	Total ≥ 70 (n = 27)	*p*-Value *
Mean Length of Stay (days, SD)	8.5 (2.1)	9.6 (2.3)	8.1 (2.2)	9.2 (2.4)	0.118
Received Structured Rehab, n (%)	12 (85.7)	16 (76.2)	14 (93.3)	20 (74.1)	0.176
Readmission Within 3 Months, n (%)	1 (7.1)	3 (14.3)	0 (0.0)	2 (7.4)	0.332
Follow-Up Compliance Rate (%) *	88.3 (9.2)	84.7 (10.1)	90.2 (8.4)	85.2 (9.6)	0.293

* SD of compliance percentage; Outcomes did not differ by rehab completion (*p* > 0.05).

## Data Availability

Data availability is subject to hospital approval.

## References

[1] Kanis J.A., Odén A., McCloskey E.V., Johansson H., Wahl D.A., Cooper C. (2012). A systematic review of hip fracture incidence and probability of fracture worldwide. Osteoporos. Int..

[2] Johnell O., Kanis J.A. (2006). An estimate of the worldwide prevalence and disability associated with osteoporotic fractures. Osteoporos. Int..

[3] Katsoulis M., Benetou V., Karapetyan T., Feskanich D., Grodstein F., Pettersson-Kymmer U., Eriksson S., Wilsgaard T., Jørgensen L., Ahmed L.A. (2017). Excess mortality after hip fracture in elderly persons from Europe and the USA: The CHANCES project. J. Intern. Med..

[4] Cooper C., Cole Z.A., Holroyd C.R., Earl S.C., Harvey N.C., Dennison E.M., Melton L.J., Cummings S.R., Kanis J.A., IOF CSA Working Group on Fracture Epidemiology (2011). Secular trends in the incidence of hip and other osteoporotic fractures. Osteoporos. Int..

[5] Papaioannou A., Morin S., Cheung A.M., Atkinson S., Brown J.P., Feldman S., Hanley D.A., Hodsman A., Jamal S.A., Kaiser S.M. (2010). 2010 clinical practice guidelines for the diagnosis and management of osteoporosis in Canada: Summary. CMAJ.

[6] Peeters C.M., Visser E., Van de Ree C.L., Gosens T., Den Oudsten B.L., De Vries J. (2016). Quality of life after hip fracture in the elderly: A systematic literature review. Injury.

[7] Panula J., Pihlajamäki H., Mattila V.M., Jaatinen P., Vahlberg T., Aarnio P., Kivelä S.L. (2011). Mortality and cause of death in hip fracture patients aged 65 or older: A population-based study. BMC Musculoskelet. Disord..

[8] Hung W.W., Egol K.A., Zuckerman J.D., Siu A.L. (2012). Hip fracture management: Tailoring care for the older patient. JAMA.

[9] Smith T., Pelpola K., Ball M., Ong A., Myint P.K. (2014). Pre-operative indicators for mortality following hip fracture surgery: A systematic review and meta-analysis. Age Ageing.

[10] Freedman V.A., Spillman B.C. (2014). Disability and care needs among older Americans. Milbank Q..

[11] Segev-Jacubovski O., Magen H., Maeir A. (2019). Functional Ability, Participation, and Health-Related Quality of Life After Hip Fracture. OTJR.

[12] World Health Organization (1996). WHOQOL-BREF: Introduction, Administration, Scoring and Generic Version of the Assessment.

[13] Ware J.E., Sherbourne C.D. (1992). The MOS 36-item short-form health survey (SF-36). I. Conceptual framework and item selection. Med. Care.

[14] Taylor N.F., Rimayanti M.U., Peiris C.L., Snowdon D.A., Harding K.E., Semciw A.I., O’Halloran P.D., Wintle E., Williams S., Shields N. (2024). Hip fracture has profound psychosocial impacts: A systematic review of qualitative studies. Age Ageing.

[15] Ouellet J.A., Ouellet G.M., Romegialli A.M. (2019). Functional outcomes after hip fracture in independent community-dwelling patients. J. Am. Geriatr. Soc..

[16] Qin H.C., Luo Z.W., Chou H.Y., Zhu Y.L. (2021). New-onset depression after hip fracture surgery among older patients: Effects on associated clinical outcomes and what can we do?. World J. Psychiatry.

[17] Zhao L., Zhao X., Dong B., Li X. (2024). Effectiveness of home-based exercise for functional rehabilitation in older adults after hip fracture surgery: A systematic review and meta-analysis of randomized controlled trials. PLoS ONE.

[18] Parsons N., Griffin X.L., Achten J., Chesser T.J., Lamb S.E., Costa M.L. (2018). Modelling and estimation of health-related quality of life after hip fracture: A re-analysis of data from a prospective cohort study. Bone Joint Res..

[19] Beaupre L.A., Binder E.F., Cameron I.D., Jones C.A., Orwig D., Sherrington C., Magaziner J. (2013). Maximising functional recovery following hip fracture in frail seniors. Best Pract. Res. Clin. Rheumatol..

[20] Welsh A., Hanson S., Pfeiffer K., Khoury R., Clark A., Grant K., Ashford P.A., Hopewell S., Logan P.A., Crotty M. (2024). Facilitating the transition from hospital to home after hip fracture surgery: A qualitative study from the HIP HELPER trial. BMC Geriatr..

[21] Mardare I., Furtunescu F.L., Bratu E.C. (2019). Measuring health related quality of life—Methods and tools. Acta Med. Transilv..

[22] Radu M.C., Armean S.M., Chivu L.I., Aurelian J., Medar C., Manolescu L.S.C. (2025). Assessing the Quality of Life of Pregnant Women in Romania: Socioeconomic, Health, and Obstetric Factors and the Validation of the WHOQOL-BREF Instrument. Nurs. Rep..

[23] Ionescu C.E., Popescu C.C., Codreanu C. (2025). Impact and Prevalence of Depression and Anxiety in Rheumatoid Arthritis-A Cross-Sectional Study with Self-Reported Questionnaires. J. Clin. Med..

[24] Cotiga A.C., Zanfirescu Ş.A., Iliescu D., Ciumăgeanu M., Gotca I., Popa C.O. (2023). Psychometric Characteristics of the Romanian Adaptation of the GAD-7. J. Psychopathol. Behav. Assess..

[25] Haddad B.I., Abu Ali M., Alashkar O., Jamos D., Alnaser I., Qambar O., Aburumman R., Altarawneh D., Karam A.M., Alshrouf M.A. (2024). Quality of Life After Hip Fracture Surgery in the Elderly: A Cross-Sectional Study. Cureus.

[26] Amarilla-Donoso F.J., Roncero-Martin R., Lavado-Garcia J.M., Toribio-Felipe R., Moran-Garcia J.M., Lopez-Espuela F. (2020). Quality of life after hip fracture: A 12-month prospective study. PeerJ.

[27] Hammer A., Ljungberg K., Bohman T., Andersson Å.G. (2022). Description and comparison of postoperative functioning of patients with hip fracture 2018 and 2008 at the Örebro University Hospital—A comparative cross-sectional study. BMC Geriatr..

[28] Kajos L.F., Molics B., Than P., Gőbel G., Elmer D., Pónusz-Kovács D., Csákvári T., Kovács B., Horváth L., Bódis J. (2024). Comparative analysis of the quality of life regarding patients who underwent hip replacement in public versus private hospitals in Hungary. Sci. Rep..

[29] Deutschbein J., Lindner T., Möckel M., Pigorsch M., Gilles G., Stöckle U., Müller-Werdan U., Schenk L. (2023). Health-related quality of life and associated factors after hip fracture. Results from a six-month prospective cohort study. PeerJ.

[30] Loggers S.A.I., Willems H.C., Van Balen R., Gosens T., Polinder S., Ponsen K.J., Van de Ree C.L.P., Steens J., Verhofstad M.H.J., Zuurmond R.G. (2022). Evaluation of Quality of Life After Nonoperative or Operative Management of Proximal Femoral Fractures in Frail Institutionalized Patients: The FRAIL-HIP Study. JAMA Surg..

[31] Kalmet P.H.S., de Joode S.G.C.J., Fiddelers A.A.A., Ten Broeke R.H.M., Poeze M., Blokhuis T. (2019). Long-term Patient-reported Quality of Life and Pain After a Multidisciplinary Clinical Pathway for Elderly Patients with Hip Fracture: A Retrospective Comparative Cohort Study. Geriatr. Orthop. Surg. Rehabil..

[32] de Abreu E.L., de Oliveira M.H. (2015). Evaluation of the quality of life of patients undergoing hemiarthroplasty of the hip. Rev. Bras. Ortop..

